# Orthoptic Home Visits for Stroke Survivors: Results from a UK Professional Practice Survey

**DOI:** 10.22599/bioj.134

**Published:** 2019-06-27

**Authors:** Kerry Hanna, Fiona Rowe

**Affiliations:** 1 University of Liverpool, GB

**Keywords:** Home visits, orthoptics, stroke, vision, domiciliary care

## Abstract

**Aim:**

Orthoptists are perhaps the only allied health profession without a standard home visits service in the UK, although it could arguably be of benefit to many orthoptic patients. The aim of this survey was to identify whether home visits are being offered, or have the potential to be offered, within the orthoptic profession.

**Method:**

A survey of the orthoptic professional body (BIOS) for the UK and Ireland was developed and data collected between January and March 2016. Descriptive analysis was used to report the quantitative findings. A thematic analysis approach was undertaken for the written responses within the free-text boxes of the survey.

**Results:**

461 BIOS members responded to the survey (response rate of 30.7%). Ten hospital sites (3.7%) reported offering home visits, and 444 members (96.3%) reported that they do not offer home visits, with little desire or perceived need for such a service. Only certain patients reportedly meet requirements for an orthoptic home visit, including those unable to attend the hospital due to poor health, transport issues, reduced cognition, stroke and learning difficulties. Implementation barriers were reported including staff safety, assessment quality and cost.

**Conclusion:**

Home visits are infrequently conducted within the orthoptic profession. However, where offered, certain patient groups were suggested to benefit from this service when they cannot attend hospital and thus, home visits could present a viable means of providing equitable visual care. Future research is required to explore orthoptic home visits compared to other forms of rehabilitation, and address concerns from the orthoptic professional body.

## Introduction

Stroke-related visual impairment is a well-documented post stroke sequelae with new onset visual impairment affecting about 60% of acute stroke survivors (Rowe et al. 2019) Where visual impairment is identified, follow-up after discharge from hospital stroke units is typically required in outpatient eye clinics. However, many stroke survivors cannot be followed up in hospital for various reasons due to their stroke and/or visual impairments, including transport difficulties, being too unwell and forgetting about appointments due to various additional ongoing outpatient appointments. Some stroke survivors request home visits in order to overcome this issue.

An international systematic review described the benefits and barriers of home visits following a stroke (Hillier & Inglis-Jassiem 2010), although little has been discussed specifically for home visits conducted in the UK and Ireland. There was an overall favour for home-based rehabilitation up to six months post discharge (Hillier & Inglis-Jassiem 2010). Benefits include reductions in cost (Young & Forster 1991) and in-patient hospital stay (Anderson et al. 2000), along with increased physical independence and reduced mortality (Young & Forster 1991;^1992^; Mayo 2000). Furthermore, stroke survivors reported a preference for home-based rehabilitation, or domiciliary therapy, as it is more convenient, allows for better understanding of their therapy (Low, Roderick & Payne 2004; Weiss et al. 2004) and offers them more clinician time per session (Roderick 2001). Langhorne (1999) further stated that domiciliary care services after stroke provide equivalent or better patient outcomes in the home, at a lower cost, and is preferred by patients and carers.

The need for home visits amongst the visually impaired population specifically has been discussed. Lederer (1982) reported that geriatric optometry patients would struggle to comprehend instructions for the use of low vision aids at home. Therefore, to accurately assist these patients, a domiciliary visit was required where lighting and magnifiers could be adjusted in their home environment, with further follow-up visits regularly needed. Lederer (1982) acknowledged that these visits can be time consuming and laborious, however they are usually the only acceptable means of prolonging the patients’ independent lifestyle and without them the outcome for these patients is often low.

However, the benefits of domiciliary care have been disputed and these concerns should be addressed where possible when considering implementing this service. Many of these studies reported a lack of benefit, as opposed to negative consequences of home visits (Langhorne et al. 1999; Holmqvist et al. 1998). The Cochrane review of alternative stroke services to avoid hospital admission concluded a lack of evidence to support or discourage home-based care following stroke, (Langhorne et al. 1999) as no statistically significant differences were reported between patient and carer outcomes following either home or hospital care. The trials identified in this review were considerably heterogeneous and so, it was not possible for the authors to draw accurate conclusions.

Furthermore, some studies found an unclear benefit from home visits, (Holmqvist et al. 1998; Lederer 1982; van Haastregth et al. 2000; van Haastregth et al. 2000) while some reported poorer outcomes of stroke survivors receiving domiciliary care, although these were not statistically significant (Gladman & Lincoln 1994). The authors postulate that these findings apply to the older, frailer group of stroke survivors who perhaps fare better in outpatient clinics (Gladman & Lincoln 1994), as independence may be preserved if encouraged to travel to hospital. Moreover, there are varied reports on the impact of domiciliary visits on carers’ mental health with some studies reporting a reduction in carer strain and improved insight into the patients’ needs (Anderson et al. 2000; Wottrich, von Koch & Tham 2007) while others conversely report an increased risk to caregivers’ mental health (Anderson et al. 2000; Low, Roderick & Payne 2004). A mixed model approach to include both domiciliary and out-patient hospital appointments may address both sides by providing staff with the educational opportunities from community settings and respite opportunities from day hospitals (Low, Roderick & Payne 2004).

None of these studies include orthoptic care as part of the home-based rehabilitation as this does not yet appear to have been investigated. Research suggests that community based rehabilitation may only be suitable for those who decline hospital admission or where hospital admission is not appropriate (Geddes & Chamberlain 2001).

Therefore, the aims of this survey were to investigate whether orthoptists in the UK and Ireland currently provide a home visit service, whether it is considered a viable or necessary service and if so, which patients specifically would benefit from home visits.

## Methods

### Development of the survey

**Figure 1:**
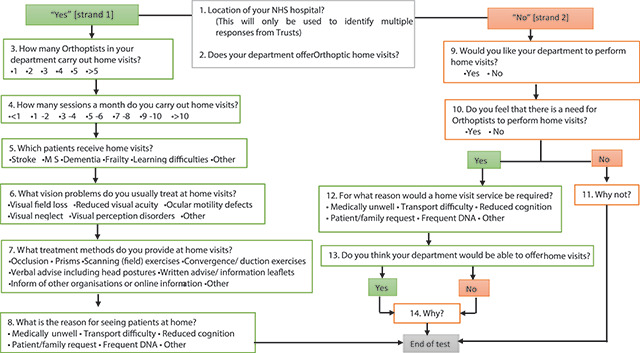
Flow diagram showing the question pathway for the survey on orthoptic home visits.

A web-based survey was developed through Survey Monkey.^460^ The online survey is the method of choice to quickly obtain vast amounts of data accurately, it is are relatively inexpensive and eliminates the risk of error as manual data entry is not required.^461^ The questions followed recommendations of using a variety of closed and open questions.^425^ Questions were kept concise with additional free-text boxes for comments to encourage all responders to complete the questionnaire (Figure 1). The survey was contained to a maximum of 10 minutes, which was advertised before the survey commenced. If the responders were already providing home visits, they were asked to report on which patients they see, how often they see them and what assessment and management options they provide. If they were not currently providing home visits, they were asked why this was the case and if their department would consider providing this service in the future. The survey was classed as a service evaluation under Health Research Authority guidelines (NHS Health Research Authority 2013) and thus, formal research ethics approval was not required, although ethical considerations were applied to the survey design and distribution. In general, a survey response of at least 10-15% is deemed acceptable (Fryrear 2015), thus a sample size of no less than 15% response rate was selected as a representation of the professional body.

### Survey distribution

The survey was emailed to all orthoptists registered with the professional body, the British and Irish Orthoptic Society (BIOS), between January and March 2016. The hospitals listed as the place of work for each orthoptist who completed the survey were mapped in order to identify whether or not an even spread of responses was achieved across the UK and Republic of Ireland. A polite reminder email was sent to the orthoptists at four-week intervals encouraging them to respond to the survey.

### Survey analysis

The results of the survey were exported to Microsoft Excel for descriptive analysis of the quantitative findings. A thematic analysis approach was undertaken for the written responses in the free-text boxes of the survey. These brief survey answers were exported into a Microsoft Word document before comments were coded, line-by-line, and analysed using the NVivo 10 software package (QSR International Ply. 2012).

## Results

### Strand 1 survey responses

A total of 461 BIOS members, from 142 hospital sites, responded to the survey out of approximately 1500 orthoptists who are registered with the professional body, eliciting an overall response rate of approximately 30.7%. Thirty-three did not complete the entire survey and dropped out at different stages but their results up until the point of drop-out were recorded and analysed.

### Provision of orthoptic home visits

Of 461 responders, 444 (96.3%) answered ‘no’ to the question of whether their department offered home visits for any patient group, and only 17 (3.7%) answered ‘yes.’ It should be noted that the latter reflects several responders from the same orthoptic service and not from 17 different hospitals or NHS Trusts. After analysis of the 17 individual responses from these sites, it was apparent that a total of 10 hospital sites across the UK reported performing orthoptic home visits. Of the 17 responders who stated that their department offers home visits, 13 answered question three and the subsequent questions of this strand of the survey, the responses of which are show in Table 1. Overall, no more than three orthoptists carry out home visits in any one orthoptic department, and very few visits are required with no more than two being undertaken per month.

**Table 1:** Responses to questions from Strand 1 of survey – orthoptists offering home visits.

Survey questions	Responses provided (from the 13 responders)	Proportion of responders (n = responders)

**How many Orthoptists in your department carry out home visits?**	1 orthoptist 2 orthoptists 3 orthoptists	n = 6, 46.1% n = 5, 38.5% n = 2, 15.4%
**How many sessions a month do you carry out home visits?**	1–2 sessions <1 session	n = 8, 61.5% n = 5, 38.5%
**Which patients receive home visits?**	Stroke Specific learning difficulties Frailty Agoraphobia Parkinson’s Dementia MS Other	n = 5, 38% n = 5, 38.5% n = 0 n = 0 n = 2, 15.4% n = 2, 15.4% n = 1, 7.7% n = 8, 61.5% *Multiple responses by each responder*
**What vision problems do you usually treat at home visits?**	VF VA OM VN VP Other	n = 5, 38% n = 7, 53.8% n = 6, 46.2% n = 5, 38% n = 6, 46.2% n = 7, 53.8% *Multiple responses by each responder*
**What treatment methods do you provide at home visits?**	Occlusion Prisms Scanning (field) exercises Convergence/duction exercises Verbal advise including head postures Written advise/information leaflets Signpost to other organisations Other	n = 5, 38% n = 5, 38% n = 3, 23.1% n = 1, 7.7% n = 8, 61.5% n = 8, 61.5% n = 6, 46.2% n = 7, 53.8% *Multiple responses by each responder*
**What is the reason for seeing patients at home?**	Medically too unwell to travel Transport difficulties Reduced cognition Frequently DNA appointments Patient/family request Other	n = 8, 61.5% n = 4, 30.8% n = 4, 30.8% n = 2, 15.4% n = 6, 46.2% n = 7, 53.8%

Legend: VA = visual actuity, VF = visual field loss, OM = ocular motility defects, VN = visual neglect, VP = visual perceptual defects, MS = multiple sclerosis.

For those who reported that their department already offers home visits, further questions were asked in order to distinguish the service already in place. Stroke patients and those with learning difficulties were reported most frequently. Additionally, where responders answered ‘other’ to question five, they included low vision patients, and ‘paediatric’ or ‘adult’ patients but did not specify a medical/orthoptic condition within these groups that would warrant a home visit.

Researchers specified that all visual deficits listed could be managed in the home, ranging from reduced visual acuity as most frequent, to field loss and neglect as least frequent. Those that selected ‘other’ for question six, failed to report additional visual impairments treated at home, but instead, used the free textbox to describe the treatments offered, which have been reported below for question seven.

The most common rehabilitation options provided were written and verbal advice and providing further information of additional services (see Table 1). Prisms and occlusion were prescribed equally with few orthoptists offering scanning and vergence exercises. The list of additional rehabilitation options reported as ‘other’ for question seven included CVI registration, the prescription of low vision aids, and accounts of combined management plans developed in coherence with a broader multidisciplinary team.

Finally, it was found that most responders assess patients who are too unwell to attend the hospital for their appointments. Where responders answered ‘other’ to question eight, they reported the benefit of assessing functional vision in the ‘real-life’ home environment, such as for patients with learning difficulties or low vision.

### Strand 2 survey responses

Four hundred thirty-four responders answered the first two questions from the second strand of the survey (one dropped out after question 1 of the survey), although only 94 continued to answer all subsequent questions (see Table 2). Overall, most responders reported no desire to begin offering home visits within their orthoptic departments. When asked to discuss possible scenarios where this service may be suitable within the orthoptic profession, the responders frequently considered medically unwell patients that are unable to travel to hospital as the main reason for warranting this service. Notably, where responders selected ‘other’ to question 12, they mainly reiterated that stroke patients would likely benefit from this service, as well as patients requiring a low vision assessment in the home setting.

**Table 2:** Responses to questions from Strand 2 of survey – orthoptists not offering home visits.

Survey questions	Responses provided	Proportion of responders (n = responders)

**Would you like your department to perform home visits?**	Yes No	n = 77, 17.7% n = 357, 82.3% (from 434 responders)
**Do you feel that there is a need for Orthoptists to perform home visits?**	Yes No	n = 97, 22.4% n = 337, 77.6% (from 434 responders)
**For what reason would a home visit service be required?**	Medically too unwell to travel Transport difficulties Reduced cognition Frequently DNA appointments Patient/family request Other	n = 85.7, 88.3% n = 47, 47.9% n = 43, 44.7% n = 22, 22.3% n = 22, 22.3% n = 32, 33.0% (from 97 responders)
**Do you think your department would be able to offer home visits?**	Yes No	n = 27, 29% n = 67, 71% (from 94 responders)

**Figure 2:**
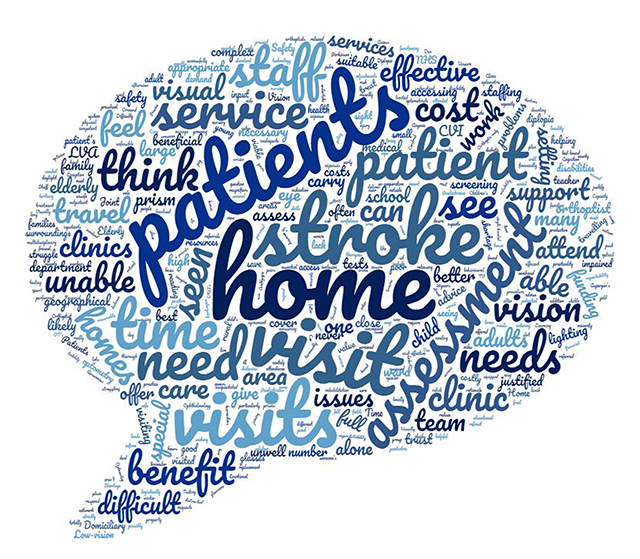
A word cloud displaying the qualitative responses from the survey responders.

For the responders answering the second strand of the survey, free-text boxes offered further explanation for their responses (questions 11 and 14), from which thematic analysis was used to explore their reasoning for whether they would like their department to implement a home visits service, and why they felt there was/was not a need for such a service. Figure 2 displays the free-text qualitative responses from the survey responders.

### Barriers to conducting orthoptic home visits

#### Staffing barriers

Staffing issues were a main barrier identified from the analysis in preventing an orthoptic home visits service, which was apparent through both the respondent’s descriptions of current job constraints, and through their language used, which expressed hesitation, and at time, astonishment, to the implementation of such services. Below, the quote from R215 uses punctuation to insinuate disbelief at the prospect of such services, with the addition of their years in practice to further strengthen their argument.

R215: ‘During my 36 years as an Orthoptist I have never had a request for a home visit!’

R356: ‘It has never been a desire in our department to do home visits.’

Moreover, the response from R356 (above) implies a certain level of control in implementing new services, dependent on the staff’s desire, and not necessarily the patients. Therefore, it appears that implementing new services requires more than just identifying a patients’ needs: it also requires convincing a workforce to partake in the new service.

Descriptions of departmental constraints were further offered, such as high transport costs to send orthoptists to patients’ homes. Orthoptists appeared under pressure to meet current hospital demands due to staff shortages and heavy workloads, and fears of further hindering their current service outweighed the benefit of conducting home visits. One responder stated that if staffing numbers were sufficient then home visits could be possible.

R273: ‘I think if it were required and it was done with all the correct policy and procedure then it should be considered. We do not have the staffing at present to offer such a service.’

Moreover, many orthoptists raised concerns over staff safety in entering patients’ homes if they were to conduct a home visit. One respondent noted that her age and gender played a role in her concern over safety when entering a patient’s home, causing a barrier to conducting home visits.

R451: ‘I feel it may be unsafe as a young female to be entering a person’s home, potentially alone, to provide a home visit.’

#### Unsuitable patient barriers

Further barriers identified considered the suitability of patients, and more specifically, that paediatric patients would not be suitable for a home visit service. Notably, no survey respondent reported that adult patients, or specifically stroke patients, would be unsuitable for this service. The reason for why paediatric patients were deemed unsuitable, was due to the array of additional visual services that they are required to attend alongside the orthoptist, which cannot be performed at home.

R365: ‘It is not an appropriate model for the clinical population seen at [hospital name] as they are all tertiary referrals and need to see other clinicians such as doctors/electrophysiology/optometry at the same appointment.’

#### Barriers to high-quality services

In addition to unsuitable patients, reports included the unsuitability of the home environment as a barrier to conducting orthoptic home visits. Some responders stated that the home environment could result in poor repeatability of orthoptic assessments and therefore, raised concerns that the orthoptic assessment at home would be counterproductive if accurate findings are required to manage the patient appropriately. This was furthered by responders’ concerns over the amount of orthoptic equipment required to undergo an assessment, and the difficulty of transporting this to the patients’ homes. For these responders, it would seem the difficulties of testing patients in the home outweigh the patient benefit, or, may even produce inaccurate test results due to poor testing conditions, which could hinder the patient further.

R434: ‘Home settings may not allow for accurate assessment due to limited testing distances and lighting conditions, therefore limited repeatability.’

R364: ‘Can you imagine how much equipment would be needed! A large amount of equipment and consumables would need to be carried in and out of patients’ homes.’

Additional responders expressed concerns regarding the travel required to conduct home visits and deemed this counterproductive and costly. This appeared to be of greater concern to those orthoptists practicing in areas that encompass a wider patient catchment area, as they could not foresee how multiple patients across the area could be seen in one session.

R53: ‘[Area name] is a large county with many rural areas and so it could take a long time to travel from one home visit to the next and would not be cost effective – you would spend more time travelling than seeing the patient.’

Furthermore, some responders discussed the benefits of testing patients in hospital clinics as a factor against supporting a home visits service. These responders reported that they would be able to assess and treat a greater number of patients in a hospital clinic. Similarly, responders reported that orthoptic patients would already be present at the hospital to see other healthcare professionals, thus rendering orthoptic home visits irrelevant.

R266: ‘Generally the hospital is better equipped to assess the patient and give them the best care.’

R177: ‘They [patients] need to see other clinicians and doctors at the same appointment. There aren’t many orthoptic-only type patients.’

Several responders suggested that a complete orthoptic assessment would not be possible, regardless of whether orthoptists conducted them. As such, it was suggested other Allied Health Professions (AHPs) including domiciliary optometrists and occupational therapists, could assess and treat the visual disorders whilst on a home visit. The statement below from R218 supports earlier reports that the orthoptic assessment at home would not be of a high enough quality, and thus, another AHP would be capable of performing the basic assessment possible without the need to staff and fund a new service. Another respondent (R192) went even further to query whether an occupational therapist (OT) would already include vision in their home assessment.

R218: ‘The tests possible at home would not be a thorough orthoptic assessment and should be able to be performed by a domiciliary Optometrist.’

R192: ‘…but would that not be part of an OT assessment?’

Arguably, one orthoptist further discussed the practice of domiciliary optometrists as an established means of assessing visually impaired patients at home, but also highlighted that optometrists would not be able to appropriately undertake the orthoptic specific assessment and management (binocular vision) of these patients. This does not entirely contest the previous suggestions that orthoptists could not perform a more accurate assessment at home, however it illuminates an inequality if the patient’s needs cannot be met through current available home-based options.

R378: ‘There is a domiciliary optometry service locally and I sometimes ask them to see adults. They would not be competent in binocular vision and diplopia however.’

Lastly, the benefits of alternative, well-established, local clinics were discussed in question 12, which may address the need to see patients in the home. Several responders concurred that a home visit service may not be feasible over large geographical areas, however, travelling to local community clinics was suggested as a consensus for both sides of the argument.

R180: ‘We have 15 community orthoptic clinics close to the patient’s homes so very few need to attend a hospital assessment. This is both cost effective and convenient for staff and patients.’

### Facilitators to conducting orthoptic home visits

#### Patient-specific needs and conditions as facilitators

The type of patient, or their specific medical/orthoptic condition, may act as a ‘facilitator’ to conducting home visits, as particular conditions may warrant a home visit above others. The survey responders suggested that medically unwell patients who are unable to travel to a hospital, would benefit from this service.

R301: ‘Unwell adults may benefit [from home visits] especially if bedbound.’

Similarly, responses to question 12 considered patients that are too unwell to travel to hospital, and those with reduced cognition, to require a home visit, as this type of patient was deemed more suitable to be assessed accurately and appropriately in the home environment. Additionally, transport difficulties preventing the patient from attending hospital were reported as a further reason for conducting home visits, as well as instances where patients and/or their family members have requested such a service.

Notably, ‘stroke’ was reported frequently where responders selected ‘other’ for question 12. It was further suggested that patients with reduced mobility, and patients with reduced confidence in attending hospital (for unknown reasons) would be suitable for a home visit.

R454: ‘I could see this being beneficial for stroke patients’ rehabilitation.’

R92: ‘The only group of patients for whom home visits could be justified and have value, would be for stroke patients or severe traumatic brain injury.’

R372: ‘This service would suit patients with reduced mobility or visual impairment affecting confidence in new surroundings.’

#### Suitability of the home setting

Despite previous reports of the home setting creating a barrier to orthoptic home visits, other responders suggested the benefits of testing an orthoptic patient at home and thus encouraged the use of such a service. The above quote from R372 suggested that the home environment would be useful in assessing patients with little confidence in unfamiliar settings, such as a hospital clinic. Furthermore, R160 (below) reported that the home setting is an appropriate place to assess vision as it considers the patient’s individual requirements in real-life situations.

R160: ‘I think it would benefit those that need a low vision assessment possible, as it would then be in their own realistic environment.’

### Survey follow-up: risk assessment, policies and procedures

Several survey responders expressed concerns regarding staff safety when performing home visits. However, as a wide range of AHPs, including a small number of orthoptists, are already providing home visits across the UK, protocols and risk assessments have been put in place for each NHS Trust in order to address this issue and ensure safety is maintained. The possible risk of performing orthoptic home visits has been evaluated in this sub-section to address this concern.

Ten responders that completed the survey and reported performing orthoptic home visits inputted their email addresses at the end of the survey granting further contact by the researcher. Two responders shared their lone workers policies for conducting orthoptic home visits, as it was identified that in both cases, the orthoptic home visits were conducted by one orthoptist at a time. The policies outlined the importance of contacting the department’s receptionist to make them aware of their safety and whereabouts. Ensuring a supervisor or other member of staff is aware of the visitor’s schedule and whereabouts is crucial. Further recommendations were made to ensure orthoptists inform an external person of their location, who they are visiting, the estimated timescale of the visit and if necessary, take another staff member with them. Moreover, they must leave their mobile phone number with the department’s receptionist, phone the department when the home visit is completed and agree on a time in which the orthoptist should be contacted should they fail to phone the receptionist. If the orthoptists have not phoned the department at the planned leaving time and cannot be contacted by telephone, they have advised the receptionist to wait 20 minutes before trying again as the orthoptist may be driving. The orthoptists should then be contacted at home, or through their next of kin before contacting the police. If the orthoptist answers the phone in distress the police should be called immediately.

The lone worker policies further suggest keeping a written log of any known risks associated with patients, home settings or locations that may be visited e.g. uneven path at the patient’s home or a known high crime area. All visits should then be individually risk assessed to ensure safety and visits should be rearranged if issues have been identified with a patient or location.

Whilst on a home visit, the policies advise workers to be aware of the warning signs for potential risks or hazards. These may include recognising dangerous animals or patients or family members/carers under the influence of alcohol or drugs. Lone workers can request animals be removed to another secure area whilst assessing the patient. The orthoptist may contact any patient known to have an animal prior to the visit and request the animal is kept in a separate room for the duration of the visit.

Furthermore, safety should be maintained if travelling by car in cases where the car may breakdown, equipment may be left in the car or where the worker feels unsafe in the car. It has been suggested that routes are planned carefully and ensure appropriate fuel is in the car. Valuables and equipment should be locked in the boot and out of sight when leaving the car and a torch, mobile phone and map kept in the car when performing a home visit. Further information regarding what to do if the worker feels unsafe whilst driving to a home visit destination is included in the lone worker policy.

#### Responding to risk on home visits

Although these methods can effectively help prevent dangerous scenarios occurring, professionals should be trained in what to do if such dangerous situations arise. NHS Trusts provides violence and aggression training for staff and can offer further self-defence training to help workers identify and cope with rare situations where their safety may be compromised (Beder 1998). The policies outlined the importance of ensuring staff attend risk management training if working alone. If an incident occurs, it is essential that visitors remove themselves from the situation and formally report the incident immediately. Reporting incidents aids development of effective interventions and strategies to enhance safety while performing home visits (Campbell 2016).

The policies further outlined what orthoptists should do if an incident occurs. All incidents of theft or assault should be reported to the police and a crime reference number obtained and added to an incident report. The line manager must ensure the incident report is submitted. Lone workers can be provided with personal attack alarms and should be used in the same way as clinic room panic buttons.

## Discussion

Overall, researchers found that most orthoptic departments do not provide a home visits service. No previous orthoptic research of this type has been conducted previously for direct comparison. For the majority of orthoptic departments without a home visit service, these orthoptists do not consider it is a necessary or feasible service, mostly due to a perceived lack of need for home visits, alongside reservations of cost, staffing, and safety.

The responders reported that only certain patients would meet requirements for an orthoptic home visit. This included those unable to attend the hospital due to poor health, transport issues or reduced cognition. Specific aetiologies may include adult patients with stroke, learning difficulties and reduced mobility, or children with learning difficulties requiring functional visual assessments.

Treatment methods identified as most suitable for home visits include advice options, prism fitting for adults and occlusion therapy for children. This concurs with the current literature that clinicians must identify those patients who would benefit most from home visits as there are some groups who would be better treated in a hospital setting (Gladman, Lincoln & Barer 1993). However, as there has been no published literature on the use of orthoptic home visits, future research is required in order to establish the effectiveness of domiciliary care in these groups, in order to inform clinicians of the potential benefit of this service.

Several responders further reported concerns over unsuitable testing conditions within patients’ homes. However, it could be argued that for patients on acute wards, such as stroke, this is often the case anyway. Furthermore, many responders conveyed the potential difficulty of bringing all necessary equipment into homes and the predicted expense of purchasing additional equipment. Again, in cases of ward assessments, a small case of equipment is usually all that is required for transporting essential tests and treatment options (Rowe 2009). Orthoptists across the UK carry similar bags of equipment when conducting preschool vision screening assessments (Alexander 1992; Ciner et al. 1999; Berg 2006). and orthoptists should be reassured that although the cost of some additional items may be required, the overall equipment should not be too cumbersome to bring on home visits.

Many orthoptists not currently performing home visits raised concerns over staff safety when entering a patient’s home. Concerns regarding staff safety whilst performing home visits for a range of professions has been acknowledged in the current literature,(Hillier & Inglis-Jasiem 2010 & Beder 1998) and it has been advised that visitors avoid known unsafe neighbourhoods and plan travel routes before departing (Beder 1998). Moreover, it has been reported that if the visitor is distracted by safety concerns then the quality of assessment is compromised (Beder 1998). However, in instances where clinicians are working alone in the patient’s home setting, various methods to address these concerns have been suggested and should be followed to ensure security and protection for staff. After contacting those departments with an established home visits service, it was revealed that these orthoptists worked alone in to conduct home visits but followed policies and procedures to ensure staff safety is maintained, as is the case with the many other AHPs performing home visits. Procedures such as ensuring others are aware of the visitor’s whereabouts; regularly checking in with others whilst performing the home visit; avoiding unsafe areas and planning the route before travelling can help prevent risk. Providing staff with the appropriate training can further enhance their safety if met with risk, whilst reporting incidents can improve the service for future visits.

Moreover, it should be noted that clinical orthoptists often work alone in individual clinic rooms and face the same risks as those entering a patient’s home to do an orthoptic assessment. The lone worker policies acknowledged this risk both in the clinical setting, during home visits and outside of work hours if undertaking administrative duties alone and urges orthoptists to be vigilant at all times. Orthoptists should translate these skills used on a day to day basis to their work in a patient’s home and follow the same procedures as they would if faced with violence or aggression in the clinic room. Furthermore, the possibility of conducting orthoptic home visits alongside other AHPs, as is already standard practice in non-eye-based community care (Langhorne et al. 1999), could further tackle the concerns discussed regarding staff safety and should be explored in future research.

In addition, telemedicine could be explored as an alternative to home visits, where orthoptic patients cannot attend hospital and/or the orthoptists are unable to conduct a home visit. A Cochrane review (Flodgren 2015) reported effective uses of telemedicine to include monitoring of a chronic condition, provision of treatment or rehabilitation, for example stroke rehabilitation, education and advice for self-management, specialist consultation, post-operative assessment after minor operations, and screening for depression or angina. Although these uses of telemedicine may not directly relate to orthoptics, it is possible that treatment monitoring, such as Fresnel prim-wear, could be supported through tele-methods and should be explored in future research. However, complications including, but not limited to, speech and language difficulties following stroke, may limit the viability of this service where patients cannot communicate over the phone (Mashima & Doarn 2008). Specific research would be required to ascertain the feasibility of conducting visual assessments via telemedicine, compared to other forms of community care including home visits, as this is currently unreported in the literature.

## Limitations

The majority of responses came from English hospital sites, limiting the potential generalisability of the views to the entire UK and Irish orthoptic profession.

## Conclusions

The findings from this survey suggest that home visits are not required for the majority of patients and only few visits would be needed per Trust. However, for the minority of patients who require this service (including stroke patients, patients with reduced mobility, cognition, confidence and learning disabilities), home visits could be greatly beneficial. The survey identified barriers to providing home visits, which included concern that the assessments would not be performed accurately. However, the responses from orthoptists already providing home visits (albeit small numbers) felt that one of the primary reasons for providing this service was that the visual assessments were performed more accurately when patients were in their home environment. Although several orthoptists suggested that a non-eye trained clinician could undertake the assessment and management of these patients during domiciliary visits, this should be considered with caution, a risk of missed or misdiagnoses of visual impairments that should be avoided at all costs. Furthermore, in cases where home visits are unequivocally impossible due to cost or staff shortages, an increase in community clinics was suggested to help address both the patients’ and clinicians needs.
